# The cell line ontology-based representation, integration and analysis of cell lines used in China

**DOI:** 10.1186/s12859-019-2724-6

**Published:** 2019-04-25

**Authors:** Hongjie Pan, Xiaocui Bian, Sheng Yang, Yongqun He, Xiaolin Yang, Yuqin Liu

**Affiliations:** 1 Institute of Basic Medical Sciences, Chinese Academy of Medical Sciences, Beijing, China; 2 University of Michigan Medical School, Ann Arbor, MI 48109 USA

**Keywords:** Cell line cell, Data integration, Ontology, Cell line ontology

## Abstract

**Background:**

The Chinese National Infrastructure of Cell Line stores and distributes cell lines for biomedical research in China. This study aims to represent and integrate the information of NICR cell lines into the community-based Cell Line Ontology (CLO).

**Results:**

We have aligned, represented, and added all identified 2704 cell line cells in NICR to CLO. We also proposed new ontology design patterns to represent the usage of cell line cells as disease models by inducing tumor formation in model organisms, and the relations between cell line cells and their expressed or overexpressed genes or proteins. The resulting CLO-NICR ontology also includes the Chinese representation of the NICR cell line information. CLO-NICR was merged into the general CLO. To serve the cell research community in China, the Chinese version of CLO-NICR was also generated and deposited in the OntoChina ontology repository. The usage of CLO-NICR was demonstrated by DL query and knowledge extraction.

**Conclusions:**

In summary, all identified cell lines from NICR are represented by the semantics framework of CLO and incorporated into CLO as a most recent update. We also generated a CLO-NICR and its Chinese view (CLO-NICR-Cv). The development of CLO-NICR and CLO-NIC-Cv allows the integration of the cell lines from NICR into the community-based CLO ontology and provides an integrative platform to support different applications of CLO in China.

## Background

Cell lines are important biological materials for biomedical research on studying specific cell functions, identifying biomarkers and searching new therapeutic methods for various diseases. Unfortunately, cell line misidentification and cross-contamination often occurs and affects scientific reproducibility [1]. Cell line depositories, such as American Type Culture Collection (ATCC) (http://www.atcc.org/), Coriell Cell Repositories (https://catalog.coriell.org/) and the Japanese Collection of Research Resources Bank (JCRB) (http://cellbank.nibiohn.go.jp), provide well-characterized and authenticated cells for usage in biomedical research [2].

The Chinese National Infrastructure of Cell Line Resource (NICR) (http://www.cellresource.cn/) is a non-profit, national cell line repository, constituted by 6 local repositories resources from different research institutes and with real biological cell samples for sales and an integrated unified cell line categories system, which provides quality-controlled cell lines and associated quality control service in China [3]. Similar to the Global Bioresource Center (https://www.atcc.org/), NICR is responsible for distributing cell line cells to its customers primarily in China. NICR also maintains the digital cataloging of cell line entries. We have clarified this in our revision. By now, NICR offers 2704 cell lines (not including stem cell lines). Meanwhile, NICR also devotes its energy into the normalized cell line description [4]. First, NICR has assigned an internal unique and persistent identifier for every cell line. Second, NICR has established the Chinese - English naming rules for cell lines. The complete information of a cell line in NICR includes the cell line name, a brief introduction of one cell line, the information about species, disease type, genetic modification and its original cell (tissue). Control vocabulary is required in NICR to represent the cell line information. For example, the cell line related diseases should be recorded by WHO international Classification of Diseases-10 (ICD-10). Third, NICR has constructed the annotation metadata and administrative metadata of cell line [4]. These standardization efforts make it possible to effectively integrate Chinese cell line information with the Cell Line Ontology (CLO), an international cell line standard [5].

CLO is a comprehensive community-based ontology in the area of cell lines and cell line cells, developed by following the principles (e.g., openness and collaboration) of the Open Biological and Biomedical Ontologies (OBO) Foundry [6]. CLO reuses terms from many existing ontologies, such as the Cell Ontology (CL) [7], Ontology for Biomedical Investigations (OBI) [8], and Disease Ontology [9]. CLO currently represents nearly 40,000 cell lines, whose information was extracted from resources such as ATCC [10], HyperCLDB [11], Coriell Cell lines, and Japan Riken cell lines (http://cell.brc.riken.jp/en/). CLO has been used in many biomedical databases and research projects, such as ChEMBL database [12], the Library of Integrated Network-Based Cellular Signatures (LINCS) and Cellosaurus (http://web.expasy.org/cellosaurus/). It has also mapped to other cell line nomenclatures such as the Experimental Factor Ontology (EFO) [13].

To better integrate NICR cell lines with the international cell line community, we have applied the CLO design pattern to reformat all the NICR cell lines, and added the information to CLO. We generated a Chinese and English bilingual CLO subset, called CLO-NICR, which contains all our NICR cell line cell information. Compared with the source CLO, CLO-NICR inherits a slim bilingual CLO framework extracted from source CLO (about 2050 classes), and includes the newly built 2704 NICR cell line cell classes, with more cell annotation properties added in bilingual presentation; more protein and gene classes and the tumor formation model of cell transplantation in vivo together with the related object properties were integrated to the subset to extend the original CLO design pattern. In addition, we presented a Chinese version of CLO-NICR, i.e. CLO-NICR-Cv, in MedPortal [14], a new Chinese-focused ontology portal generated as part of the recently initiated OntoChina, a newly initiative program aimed at coordinating the research, development, and applications of biomedical ontologies in China [15].

## Methods

### NICR data collection and cleaning

The physical cells of NICR are distributed in 6 individual centers in China, which are considered as the branches of NICR. The data of all these cells are collected, stored, and queriable in NICR of the Institute of Basic Medical Sciences, Chinese Academy of Medical Sciences, Beijing, China. The cell lines and their characteristics from NICR online database were extracted automatically and stored in EXCEL files, following a manually review and cleaning. It should be noted that the above data of the cells characteristics are originally recorded in Chinese. We translated the data from Chinese into English, and used the bilingual data to prepare for the construction of CLO-NICR using both languages.

### CLO mapping and collection

In NICR, the information of each cell line cell is expressed in the format illustrated as: “;SW480 [SW 480; SW-480]”, where the Chinese name is the cell type (i.e., human colon cancer cell) of the cell line SW480, followed by the cell line label SW480 and its alternative names. Based on this format, we developed a software program to automatically extract all the cell line names involved in the corresponding Chinese standardized name, such as “SW480”, “SW 480”, and “SW-480” in the former example. Then, we compared them with the labels or synonyms of all the cell line cells in CLO, and screened out the related CLO classes with the following procedure. For those cell line cells with exact mapping, the corresponding equivalent class information was extracted automatically first with a small VBA software program. For those cell line cells where we could not find exact mapping, we identified the information through manual search and evaluation based on the results identified from the NCBO BioPortal [16], Cellosaurus (https://web.expasy.org/cellosaurus/), and Ontobee [17]. The final identified ontology mapping information was gathered together and used for further processing as described below.

### Construction of the CLO subset for NICR

The CLO-NICR is aligned with and extends the framework of the international CLO ontology. With the collected CLO mapping relations in section B, we first used the Ontofox tool [18] to obtain a simplified version of CLO with top level classes and properties and the CLO cell line cell classes mapped to NICR cells, to start the generation of the basic framework of CLO-NCLR and facilitate the subsequent mapping and integration of NICR cells to CLO. The Protégé-OWL editor [19] was used for manual ontology editing.

In manual data mining of characteristics entries, a large amount of annotation information (e.g., proteins and genes expressed or overexpressed in cell line cells) was obtained. Our CLO-NICR includes extra information not covered by the original CLO framework. To incorporate many new cell line-related features into the ontology, we extended the basic CLO design pattern through discussions and communications with CLO developers and ontology experts, including Dr. He (an active CLO developer and a co-author of this paper).

Based on the obtained mapping relations and the extended CLO-NICR design pattern, the NICR-specific cell classes were built according to former mapping relations. In view of the reuse of the annotation properties for presenting of the 19 cell characteristics, a screening for exact annotation property for the above characteristics was performed, resulting in the discovery of many existing properties in CLO and other ontologies, such as “STR profile” in CLO and the general “rdfs:comment”, which can be used to directly describe the “STR” and “Comment” entries. To present the remaining cell characteristics, some novel annotation attributes were generated with a new CLO ID and extended to cover the rest entries, such as annotation property “karyotype” used to describe the number and appearance of chromosomes within the nucleus of a eukaryotic cell. By using an internally developed customized Java program based on OWL API, the properties information stored in EXCEL file and some related entities were inserted into the general CLO-NICR framework.

### Integration the mappings relation with other ontology

We used ‘rdfs:seeAlso’ annotation property from the Information Artifact Ontology (IAO) (https://github.com/information-artifact-ontology/IAO) to present equivalent mapping relations in other ontologies, and applied ‘has_broad_synonym’ annotation property to depict other mapping relations.

### CLO-NICR availability

The source code for CLO-NICR is available on the CLO GitHub website: https://raw.githubusercontent.com/CLO-ontology/CLO/master/src/ontology/CLO-NICR.owl.The CLO-NICR OWL file was also deposited in Ontobee (http://www.ontobee.org/ontology/CLO-NICR),and could be used to query on the Ontobee SPARQL Web program (http://www.ontobee.org/sparql) [17].

### MedPortal display of CLO-NICR

MedPortal (http://medportal.bmicc.cn) is a mirror of NCBO BioPortal [16] in China. MedPortal has been developed for the storage, analysis and visualization of ontologies targeted for Chinese users. To promote the open and share of CLO-NICR, we have first generated the Chinese view of the CLO-NICR (CLO-NICR-Cv) by using an internally developed Java software program. CLO-NICR-Cv contains only Chinese annotation of the cell lines in NICR. CLO-NICR-Cv was deposited into the MedPortal triple store. The information of CLO-NICR-Cv can then be displayed, retrieved, and analyzed in the MedPortal website (http://medportal.bmicc.cn/).

## Results

### Collected and processed NICR cell line data

We collected the information of the 2704 cells and 19 characteristics involved in the description of each cell in NICR. These cells were derived from 94 organisms. Among these cells are 917 hybridoma cells.

**Fig. 1:**
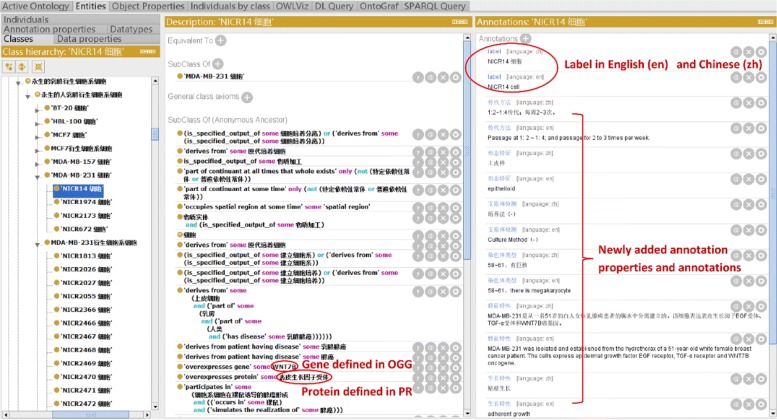
Annotation updates and enrichment in CLO-NICR. The updates and enrichment mainly came from the entities from Ontology of Genes and Genomes (OGG) and Protein Ontology (PR), and some newly added properties and annotations. The annotation properties and axioms were bilingual presented

CLO-NICR includes 19 annotation types for annotating cell line cells. Each annotation type can be described in in both English and Chinese (Fig. 1). For Chinese users, it is often preferred to read in Chinese. Therefore, we tried to annotate each CLO-NICR term with a Chinese and an English presentation respectively. For example, the English term ‘NICR cell’ can also be expressed as the Chinese term ‘NICR14 ’, both having the same ontology ID of CLO_0052087. To separate the expression of the term in these two languages, we used the W3C-specified xml:lang (https://www.w3.org/TR/xml11/). This solution is aligned with the suggestion provided by the OBO Foundry. In our case, “en” is used for English and “zh” for Chinese as shown below:

<*rdfs*:*label* *xml*:*lang*=“*en*” >*NICR*14 *cell*</*rdfs*:*label*>

<*rdfs*:*label* *xml*:*lang*=“*zh*” >*NICR*14  </*rdfs*:*label*>

The default display of English or Chinese version in Protégé can be set up by changing the Protégé Renderer preferences.

Among all CLO-NICR cells, 1234 cells have CLO mappings. Our CLO and NICR term mapping relations includes direct CLO mappings and indirect CLO mappings. As an example of the direct CLO mapping, NICR14 cell (i.e. “NICR14 ” in Fig. 1) in NICR is mapped to MDA-MB-231 cell in CLO (CLO_0007634) with exact the same cell label. In one of previous CLO paper [20], Japan cell bank cell line cell terms (e.g., term labels “RCB1648 cell” and “RCB1886 cell”) were asserted as subclasses of the corresponding CLO terms (e.g., term label with ID CLO_0003704) with the consideration that these two Japan cell bank cell line cell types may have genetic variations in their long time of passages. Using the same strategy, we asserted the NICR14 cell as a subclass for MDA-MB-231 cell (i.e. “MDA-MB-231 ” in Fig. 1) in CLO-NICR.

As an example of our indirect CLO mapping, we found that NICR2026 cell (i.e. “NICR2026 ” in Fig. 1), namely MDA-MB-231-GEP cell, as a MDA-MB-231 derived cell line in which GFP protein are stable transfected and overexpressed. We could not find the direct mapping class, MDA-MB-231 derived cell line cell in source CLO, but we could identify the indirect mappings between NICR2026 cell and MDA-MB-231 cell. So for these cases, we generated a new sibling class, such as MDA-MB-231 derived cell line cell (i.e. “MDA-MB-231 ” in Fig. 1), of the existing cell line class, such as MDA-MB-231 cell in CLO, and then added its children, such as NICR2026 cell, as a subclass of the newly built MDA-MB-231 derived cell class in Fig. 1 [5]. And for the rest 1470 cells with no CLO mapping data but some having mapping relations with other ontologies and databases, we could integrate them into general CLO framework by identifying them as the subclass of finite cell line cell, immortal cell line cell or hybridoma cell line cell owing to limited disclosed information.

### CLO ontology design pattern extension and usage

**Fig. 2:**
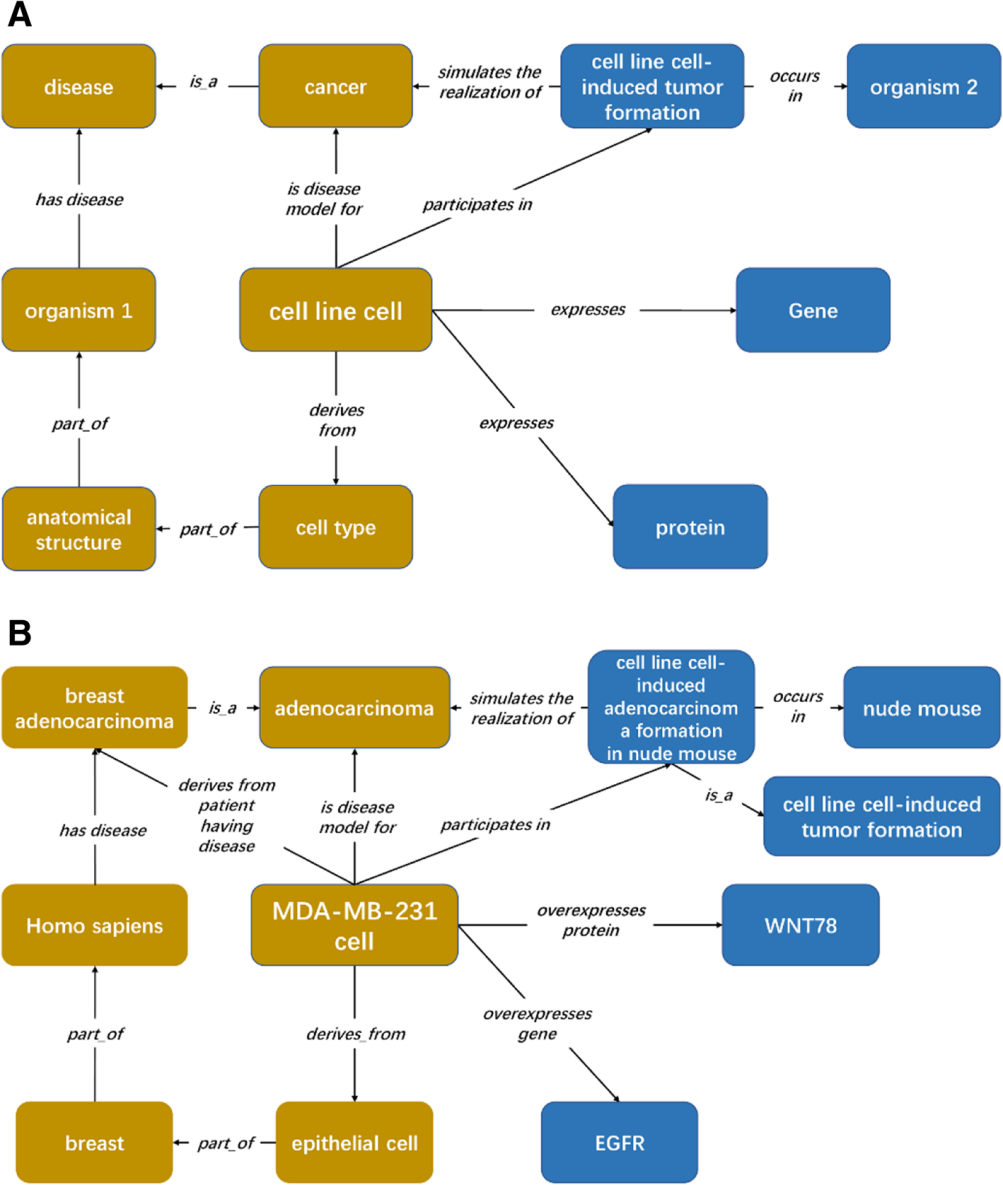
CLO-NICR design pattern and its illustration. **a** An extended general CLO design pattern. **b** An illustration of the CLO-NICR design pattern with MDA-MB-231 cell. Blocks in grown are entity and relations showed in original CLO design pattern. Blocks in blue are newly developed pattern

Depending on the original design pattern of CLO [5] we can obtain the following typical expression (Fig. 2a):

‘cell line cell’: *‘derives from’ some (‘anatomical structure’ ‘part of’ some (‘organism’ and (‘has disease’ some disease)))*

A newly published paper also introduces an extension of the CLO design pattern, where a shortcut relation ‘derives from patient having disease’ is used to indicate the relation between the organism and disease [20]. Based on this model, we can say that ‘MDA-MB-231 cell’ ‘derives from patient having disease’ ‘adenocarcinoma’ (Fig. 2b). In biomedical research, cell lines are frequently used as models for cancer research. For example, MDA-MB-231 cell has been used as model to study adenocarcinoma formation [21]. To represent such information, we can use the CLO relation ‘is disease model for’. For example, we can prepare an axiom:

Compared to these original CLO design patterns, our extended design pattern includes more semantic relations that represent how a cell line cell is used as a model to study cancer, Specifically, a cell line cell can be transplanted into an organism (e.g., mouse), and grow into a tumor under a specific experimental condition. Here, we used the organism1 to denote the cancer developed naturally in animal bodies, undergone the process of transformation of normal cell into cancer cell without any intended manual operation inference, while that for ‘organism2’ are transplant recipients for cell line cells in creating transplantation models. And the transplanted target sites in the animals are chose manually, and the recipient animals are usually model organism of no cancer formation before transplantation. And in most transplant models, the cell line cell source organism is different from the transplantation recipient organisms. In this case, the cell line cell participates in a ‘cell line cell-induced tumor formation’ process that simulates cancer formation in cell line cell manual transplantation in model organisms. To fully annotate the relation between ‘cell line cell-induced tumor formation’ and ‘cancer’, we created a shortcut relation, ‘simulates the realization of’, and the related entities can be logically represented as shown in Fig. 2a:

‘cell line cell’: *‘participates in’ some (‘cell line cell-induced tumor formation’ and ((‘occurs in’ some ‘organism’) and ‘simulates the realization of’ some ‘cancer’))*

For example, MDA-MB-231 cell can grow and develop into adenocarcinoma in the nude mouse [21]. Based on the above general design pattern, we can develop an axiom as follows:

‘MDA-MB-231 cell’: *‘participates in’ some (‘cell line cell-induced adenocarcinoma formation in nude mouse’ and ((‘occurs in’ some ‘nude mouse’) and (‘simulates the realization of’ some ‘adenocarcinoma’)))*

NICR and ATCC often provide the information of expressed or overexpressed genes and proteins in specific cell line cells. To represent the gene expression information, we imported the object property “expresses” from the Relations Ontology (RO) (http://purl.obolibrary.org/obo/ro.owl) and built two object sub-properties ‘overexpresses gene’ and ‘overexpresses protein’ to specify the overexpression of a gene and protein in cells, respectively. Here genes are represented using terms from the Ontology of Genes and Genomes (OGG) [22], and proteins using Protein Ontology (PR) [23]. For example, we generated the following axioms (Fig. 2b):

‘MDA-MB-231 cell’: *‘overexpresses gene’ some ‘WNT78’*

‘MDA-MB-231cell’: *‘overexpresses protein’ some ‘EGFR’*

### Application of CLO-NICR to NICR service measurement

Based on the user purchase records in NICR, we found top 10 bestseller cells (Table 1). Most of the cells are cancer related epithelial cells and derived from various anatomical structures. It should be noted that BV2, 3T3-L1 and 293T cells are derived from in vitro genetic operations. For example, 3T3-L1 is a cell line derived from mouse 3T3 cells. The 293T cell is a highly transfected derivative of human embryonic kidney 293 cells, and contains the SV40 T-antigen. And the related cancer derived cells line are not only involved in the study of cancer related mechanism, such as inflammatory reaction, drug resistance, cell proliferation, but also involved in the conventional research of non-cancer related mechanism. Hence, we could not specify the hotspots in cell line related cells or, but we could have a better knowledge of the frequent used cell line models in cell line related research. And for most of the NICR cell line, more typical use case is needed since that most are just in store state without any purchase record. And with the statistics, we can have a better budget forecast in manpower and material resource allocation in maintaining of all the NICR cells.

**Table 1:** TOP 10 Bestseller cells in NICR

#No.	Cells Information			
	Cell line names	Cell type	Disease	Anatomic entity
1	RAW 264.7	Monocyte	Leukemia	Blood
2	A549	Epithelial cell	Lung cancer	Lung
3	BV2	Microglia	Immortalized transformation	Central nervous system
4	CACO-2	Epithelial cell	Colon cancer	Colon
5	HepG2	Epithelial cell	Liver cancer	Liver
6	THP-1	Monocyte	Leukemia	Blood
7	MCF7	Epithelial cell	Breast cancer	Breast
8	SH-SY5Y	Epithelial cell	Neuroblastoma	Bone marrow
9	3T3-L1	Fibroblast	Immortalized transformation	Embryo
10	293T	Epithelial cell	Immortalized transformation	Kidney

**Fig. 3:**
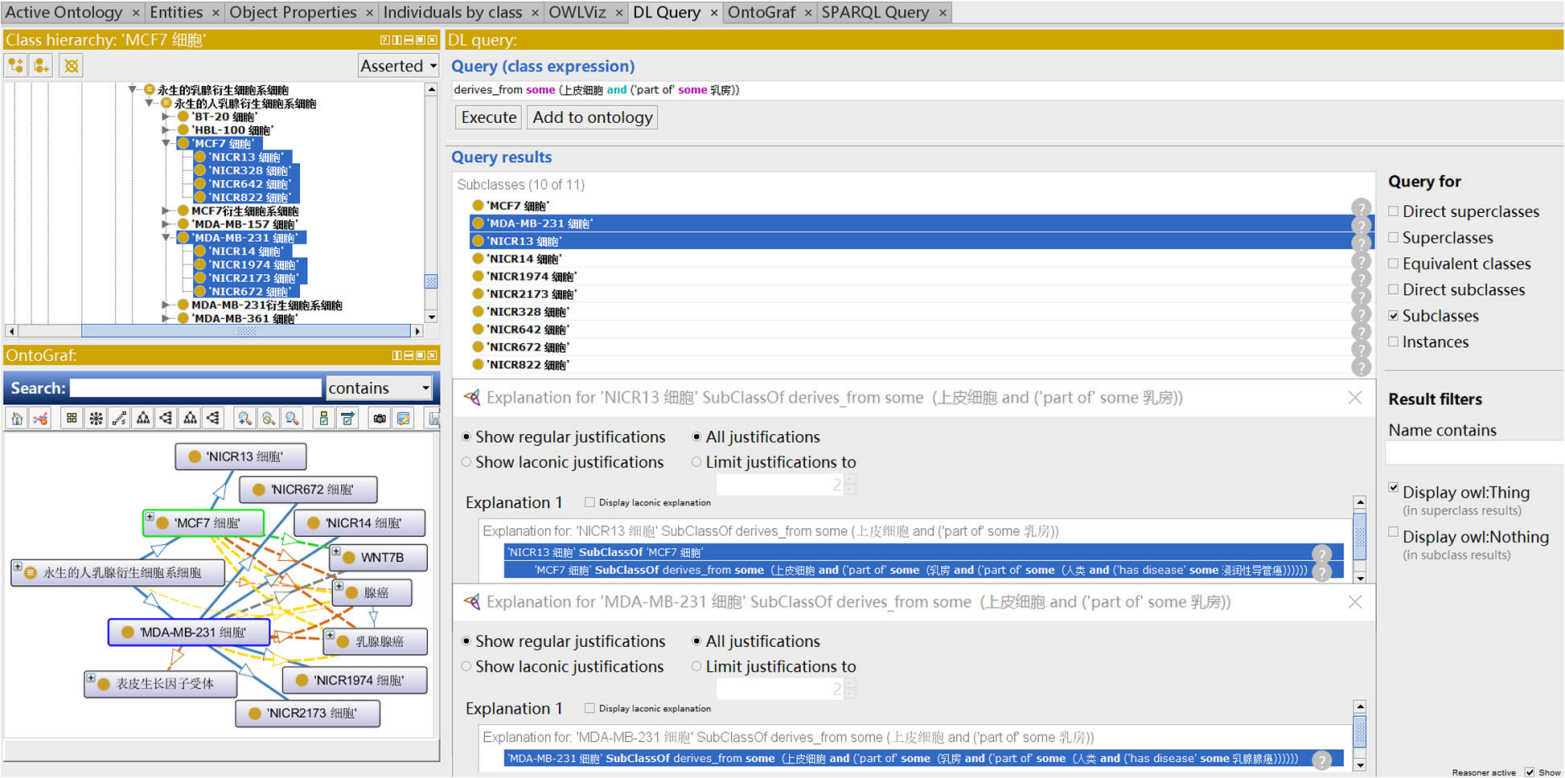
DL Query for breast derived epithelial cell. The query was performed using Protégé DL Query

And in many cases, some frequently used cell lines are cloned and stored in more than 2 repositories to benefit the users in different region. To demonstrate depository quantity and areal distribution of some frequently used cell line models in NICR, we applied a DL query on CLO-NICR under the Protégé-OWL editor platform (Fig. 3). And the MCF 7 cell, a breast derived epithelial cells, was used in the DL query. With Chinese label “” for epithelial cell and Chinese label “” for breast, the ontological axiom can be managed as follows:

*‘derives from’ some* (‘’ *and(‘part of’ some* ‘’)

This query identified 10 cell line cell types, including the original MCF7 cell in source CLO and 4 subclasses, NICR13, NICR328, NICR822, NICR642 cells in NICR, consistent with the data gathered in Table 1. Figure 3 also provides a comprehensive explanation of NICR13 cell. Specifically, this cell is derived from breast epithelial cells from a human (i.e., “” in Chinese) patient who had an invasive ductal carcinoma disease (i.e., “” in Chinese). Figure 3 also shows that this NICR13 cell is a subclass of the MCF7 cell (i.e., “MCF7 ”). Furthermore, Fig. 3 shows MDA-MB-231 cell (i.e., “MDA-MB-231 ”) and all its 4 NICR subclass cell types. Such information can also be queried using SPARQL query after the CLO-NCLR is deposited into a RDF triple store [16].

### CLO-NICR visualization in Chinese in MedPortal

**Fig. 4:**
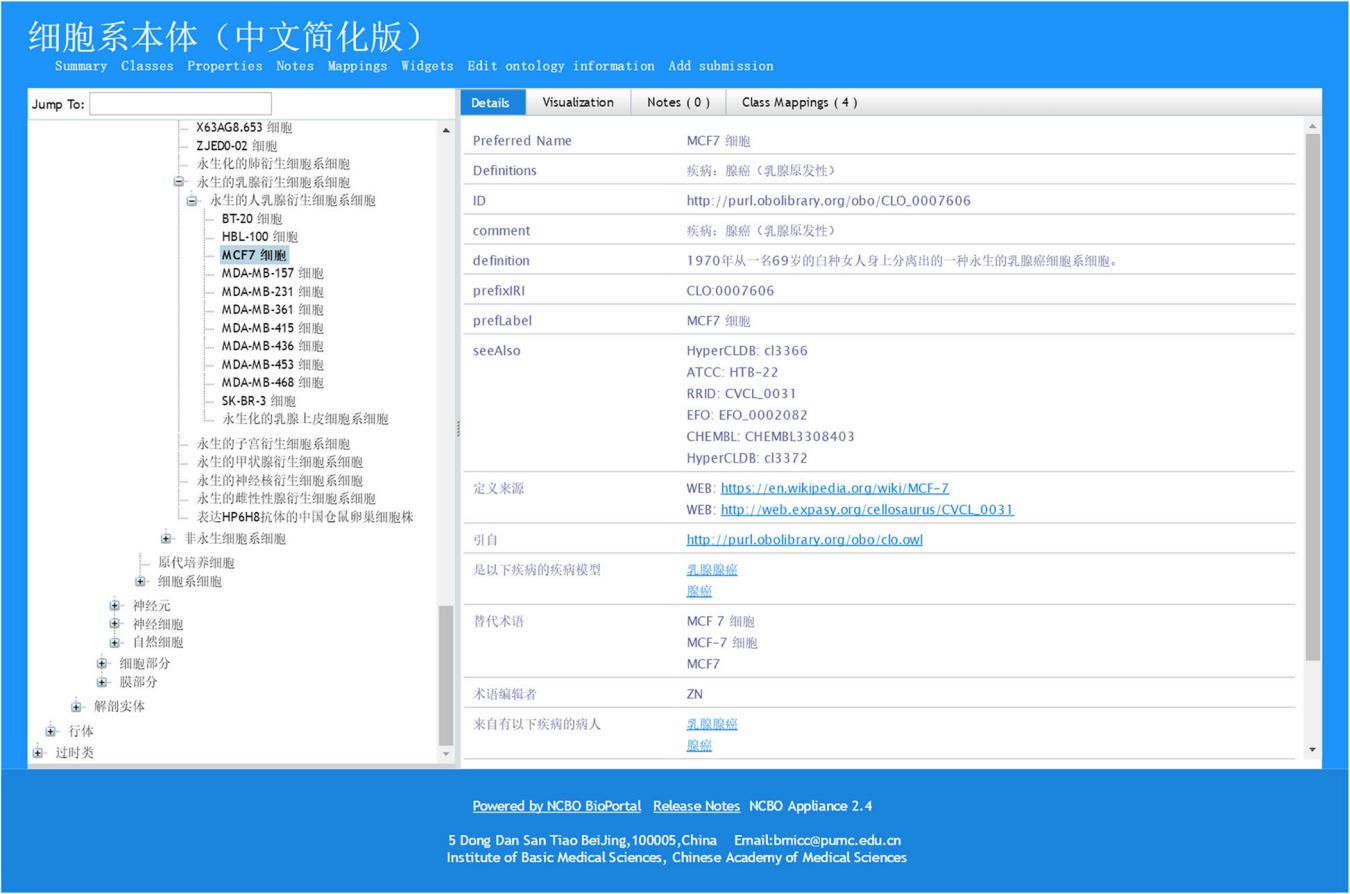
CLO-NICR views on MedPortal. Here showed some annotation properties for MCF7 cell specifically

To benefit the application of CLO-NICR and CLO-NICR-Cv, we have uploaded the CLO-NICR-Cv to MedPortal (http://medportal.bmicc.cn) to accelerate the process (Fig. 4). In order to support the visualization of Chinese ontology in MedPortal in a proper semantically rich and consistent manner [24], the labels and definitions of annotation properties in CLO-NICR were translated and refined. Users can browse online to search detailed cell line cell information, browse the cell hierarchy, and identify the relations among cells.

## Disscusion

The contributions of this study are multiple-fold. First of all, the representation of the 2704 cell line cells in NICR to CLO-NICR standardizes the commonly used cell line cells in China. The CLO-NICR framework provides us with the basis for cell related data integration and systematic analysis. The addition of the CLO-NICR also extends the coverage of CLO, and makes the cell lines commonly used in China available for standard queries and analysis. Second, this study extends the existing CLO design pattern and builds an integrative ontological framework for representing all NICR cell line cells and their detailed information. In modeling the framework of CLO-NICR, we extend the design pattern to cover new information such as the usage of cell line cells in cell transplantation for tumor generation in model organisms, and overexpressed gene and its products. The extended representation makes the cell annotation more completed and definite. In addition, CLO-NICR also includes more annotation properties and linkage information to other cell resources, e.g., ChEMBL, HyperCLDB, ATCC, Cellosaurus, and more bioinformatics databases related with cell lines. Third, we are the first to present CLO information using a bilanguage format. Considering that most of the users are native Chinese and the need for international promotion, we displayed the CLO-NICR ontology by utilizing bilingual presentation to fulfill the language need for the Chinese users. Fourth, CLO-NICR view in Chinese could be properly rendered in MedPortal, which is built by reusing the framework of NCBO BioPortal. Thus the users in China can browse, search, visualize and comment on ontologies through a user-friendly Web interface, and programmatically, via Web services. That will be helpful for promote the acceptance of CLO in China.

**Fig. 5:**
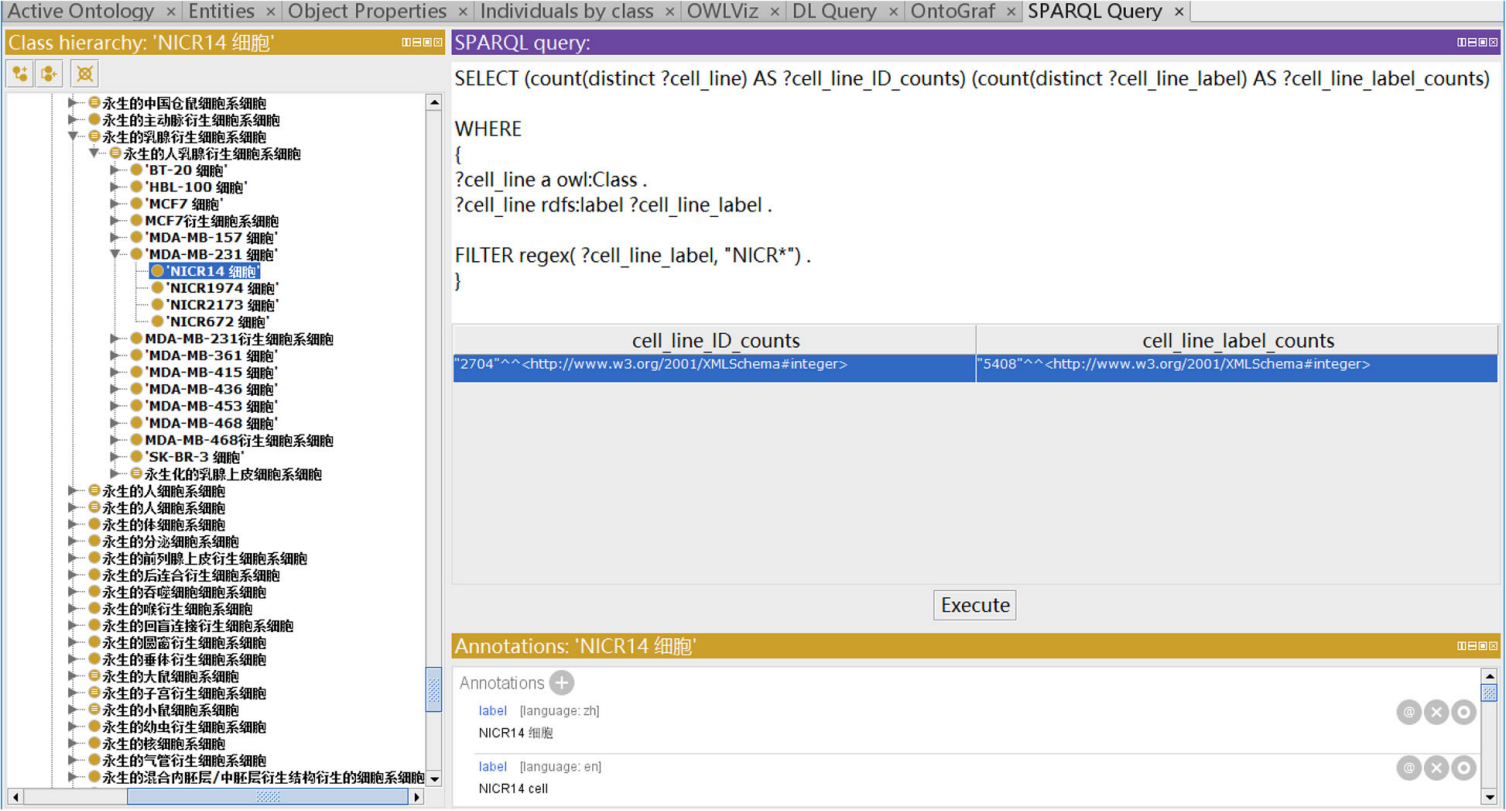
SPARQL query of the number of NICR cell lines and the corresponding cell line labels. The query was performed using Protégé SPARQL Query

We have encountered many opportunities and challenges in our cross-language axioms generation and usage. As shown in our “Results” section, we developed a strategy to make our CLO-NICR available for Chinese users by adding Chinese version of annotations such as term label (rdfs:label). Our Chinese annotated terms are also shown up in axioms. For example, the Chinese term ‘’ (i.e., cell separation) (OBI_0600037) is used in an axiom as shown in Fig. 1. In this example, this term can be expressed in Chinese as ‘’ or in English as ‘cell separation’. Overall, the usage of either English or Chinese does not affect the compatibility/equivalency of axioms when moving across different languages since either the English of Chinese version uses the same ontology ID. Our analysis shows that the axioms used with different languages are compatible and equivalent in SPARQL. This is because that ontology term ID (e.g., OBI_0600037) is used in SPARQL query. However, such a selection affects the DL query under the Protégé platform since the labels instead of ontology IDs are used in DL query expression. And when we use a SPARQL query in Fig. 5 to check the newly built NICR cell line number and the corresponding cell line label number, we found that 2704 NICR cells were integrated into the CLO-NICR with 5408 NICR cell line labels, consistent with the bilingual terms label setting for each NICR cell line cell showed in Fig. 1.

OntoChina (http://ontochina.org) is a newly initiated program in China that aims to support the development of reference ontologies, especially bilingual ontologies and Chinese translated ontologies, to establish an integrative informatics framework for productive ontology research and applications, and to promote the formation of a collaborative biomedical ontology research community. Many programs have been established under OntoChina. For example, by adopting the National Center for Biomedical Ontologies (NCBO) BioPortal technology, we set up an ontology repository (http://medportal.bmicc.cn) to accelerate the ontology application. Representing the cell lines used in China in CLO is a part of OntoChina’s mission to promote Chinese ontology development and application. OntoChina has also translated the Basic Formal Ontology [25], the most commonly used upper ontology in the OBO ontology community, into Chinese. Its final version will be available in MedPortal after another round of active peer reviewing. The related classes annotated in Chinese have been imported into the CLO-NICR-Cv version in MedPortal. We are translating several other widely used ontologies, such as the IAO and the Ontology for Biomedical Inverstigations (OBI) [26]. In addition, the OntoChina team has established a collaborative relationship with the international OBO Foundry, and more collaborative projects are anticipated in the near future.

## Conclusion

In summary, all identified cell lines from NICR are represented by the semantics framework of CLO and incorporated into CLO as a most recent update. We also generated a CLO-NICR and its Chinese view (CLO-NICR-Cv). The development of CLO-NICR and CLO-NIC-Cv allows the integration of the cell lines from NICR into the community-based CLO ontology and provides an integrative platform to support different applications of CLO in China.

## Abbreviations

ATCC: American type culture collection
CL: Cell ontology
CLO: Cell line ontology
EFO: Experimental factor ontology
IAO: Information artifact ontology
ICD-10: International classification Of disease 10
JCRB: Japanese collection of research resources bank
LINCS: NIH common fund library of integrated network-based cellular signatures
NCBO: National center for biomedical ontologies
NICR: Chinese national infrastructure of cell line resource
OBI: Ontology for biomedical investigations (OBI)
OBO: Open biological and biomedical ontologies
OGG: Ontology of genes and genomes
Ontobee: OntoChina: The China biomedical ontology consortium
PR: Protein ontology
